# Identification of a Newly Conserved SLA-II Epitope in a Structural Protein of Swine Influenza Virus

**DOI:** 10.3389/fimmu.2020.02083

**Published:** 2020-09-16

**Authors:** Massimiliano Baratelli, Sophie Morgan, Johanneke Dinie Hemmink, Elizabeth Reid, Brigid Veronica Carr, Eric Lefevre, Sergio Montaner-Tarbes, Bryan Charleston, Lorenzo Fraile, Elma Tchilian, Maria Montoya

**Affiliations:** ^1^Centre de Recerca en Sanitat Animal (CReSA), UAB-IRTA, Universitat Autònoma de Barcelona, Bellaterra, Spain; ^2^The Pirbright Institute, Surrey, United Kingdom; ^3^Innovex Therapeutics S.L., Badalona, Spain; ^4^Animal Health Department, Universidad de Lleida, Lleida, Spain; ^5^Centro de Investigaciones Biológicas Margarita Salas (CIB-CSIC), Madrid, Spain

**Keywords:** swine influenza, class II epitopes, swine, IFNγ, nucleoprotein

## Abstract

Despite the role of pigs as a source of new Influenza A Virus viruses (IAV) potentially capable of initiating human pandemics, immune responses to swine influenza virus (SwIV) in pigs are not fully understood. Several SwIV epitopes presented by swine MHC (SLA) class I have been identified using different approaches either in outbred pigs or in Babraham large white inbred pigs, which are 85% identical by genome wide SNP analysis. On the other hand, some class II SLA epitopes were recently described in outbred pigs. In this work, Babraham large white inbred pigs were selected to identify SLA II epitopes from SwIV H1N1. PBMCs were screened for recognition of overlapping peptides covering the NP and M1 proteins from heterologous IAV H1N1 in IFNγ ELISPOT. A novel SLA class II restricted epitope was identified in NP from swine H1N1. This conserved novel epitope could be the base for further vaccine approaches against H1N1 in pigs.

## Introduction

Swine Influenza is an important respiratory pig disease caused by Influenza A Virus (IAV) (1) which represents a significant problem to farming but also carries very substantial risks to human health. Domestic pigs cover an important role in the ecosystem of IAV; it is believed that they may serve as “mixing vessels” for the generation of novel reassortant influenza A virus potentially capable of initiating human pandemics (2, 3). Controlling the virus in pig populations is of great importance not only for implementing and/or improving control and surveillance programs on farms but also for preventing zoonotic infections. Thus, increasing basic knowledge of immune responses of pigs to IAV as a basis for developing novel and improved vaccines is of great importance.

Selecting the optimal antigen is a cornerstone in rational vaccine design and depending on the desired response, proper B or T cell epitopes should be carefully selected. Besides the antibody response, the T cell response is increasingly gaining more attention in the research of influenza vaccines due to its importance in protection and its ability to provide a wider breath of protection by targeting conserved domains (4). The T cell response against influenza is not fully understood in pigs (5) and even less is known about IAV T cells epitopes recognized by Swine Leucocytes Antigens (Mayor Histocompatibility Complex of pigs). T cells are considered actively involved both in clearance of the primary infection and in the control of heterologous reinfection of IAV (6). Specific CD4^+^ and CD8^+^ T cells are produced locally and systemically in pigs after IAV infection and they show a multifunction profile, cross-reactivity and memory similar to viral infections in humans (6, 7).

Reverse vaccinology is not fully developed and accurate for pigs due to the insufficient data on SLA properties on which to train and/or test algorithm for prediction of T cell epitopes. Nevertheless, pan methods have been successfully used to identify SLA epitopes of both classes (8, 9); these methods can predict T cell epitopes that bind to MHC for which experimental data are limited or not available. Therefore, empirical strategies are still crucial to generate the basic knowledge to generate novel pig specific prediction methods.

This study aimed to identify novel SLA-II epitopes of IAV in pigs by means of empirical methods. The Babraham is a large white inbred pig lineage that is 85% identical by genome wide SNP analysis (10). The matching of MHC class I and II alleles between individual animals makes them instrumental for immunological studies. Babraham's pigs were immunized in this study following the concept that inactivated vaccines are generally designed to present and promote presentation of extracellular antigens. Thus, those type of antigens should be preferentially processed toward and presented by class II SLAs. It has been shown in humans that CD4^+^ T cell responses focused mainly on NP and M1 proteins of IAV and that class II HLA epitopes are most commonly found on internal proteins (11, 12). For this reason, the specificity of the recall response produced by overlapping peptides covering the NP and M1 protein from heterologous IAV was tested using PBMC from immune Babraham's pigs and an IFNγ ELISPOT assay. In this way, we were able to identify two new SLA-II restricted epitopes in a structural protein that was conserved between IAV and could represent a potential epitope to induce protective responses against several IAV subtypes.

## Materials and Methods

### Animals

Babraham's pigs were housed at the Greenfield farm (Compton) belonging to The Pirbright Institute. Those are inbred large white pigs syngeneic at the MHC loci. Animals were *Mycoplasma Hyopneumoniae* and Porcine Reproductive and Respiratory Syndrome Virus free. They were vaccinated against Glässer disease (MSD Animal Health), Erysipelas (Porcillis Ery Injection, MSD Animal Health), Parvo Virus (Suvaxyn Parvo/E Injection, Zoetis) and received treatment for Mites and Parasites with Panomec Injection (Merial). Cohort 1: animals were 25–30 weeks old and around 39–50 Kg of weight; they were labeled B557, B558, B563, B564, B568, and B570. Cohort 2: animals were 8–9 weeks old and around 5.5–9.5 Kg of weight; they were labeled CTRL, 1 and 2. Cohort 3: animals were 2–3 years old and around 173–192 Kg of weight; they were labeled B625 and B650. All studies were carried out under UK Home Office License number 70/7505 and approved by the ethical review processes at The Pirbright Institute.

### Immunizing Antigens

Three type of antigens were selected: SpH1N1, Gripovac3 and S-Flu. The strain A/Swine/Spain/SF11131/2007 (SpH1N1) belonged to the swine lineage of IAV and in particular to the European “Avian-like” (13). This was propagated on Madin Darby Canine Kidney cells (MDCK) as described previously (13) and inactivated by UV radiations. Inactivation of the virus was confirmed as absence of formation of any cytopathic effect after 7 days incubation in a MDCKs monolayer. Integrity of the viral proteins was indirectly confirmed as consistence of Hemagglutination Units (HAU) before and after UV inactivation. Hemagglutination test was performed as described elsewhere (WHO). One dose of the immunization antigen SpH1N1 was prepared by mixing the volume corresponding to 1–3.5 × 10^7^ TCID_50_ of the inactivated viral solution with 1 ml of the adjuvant Montanide ISA 206 VG (Seppic). The second immunizing antigen solution was Gripovac3 (IDTbiologika) which is constituted by whole inactivated IAVs formulated with adjuvant. The vaccine contained Europeans strains of the swine lineage of H3N2, H1N1, and H1N2 subtypes. The third immunizing antigen was a recombinant live IAV (S-Flu) constituted by an H5 Hemagglutinin and the rest of proteins from the human IAV A/PR/8/34. This virus was generated and attenuated by suppression of HA signal sequence by Dr. Alan Townsend (University of Oxford, UK) as described in (14). One dose of S-Flu was containing 6 × 10^7^ TCID_50_.

### Pig Immunization and Sampling

The cohorts of pigs selected for the study were immunized with three different strategies implying two dose of the IAV antigens administered within a 28 days interval (Table 1). The inactivated antigens were administered intramuscularly in the front shoulder of the animals. The live antigens S-Flu was administered in the nasal cavities using a mucous administration device (Wolfe Tory Medical). Blood was collected in heparinised tubes by anterior vena cava venepuncture at 0, 7, 14, and or 21 days post boost (dpb). Peripheral Blood Mononuclear Cells (PBMCs) were obtained by density gradient centrifugation of blood, 1,200xg for 30 min over Histopaque 1.083 g/ml (Sigma). RBC were lysed by 5 min incubation on ice with Ammonium Cloride buffer (Ammonium Cloride 155 mM, Potassium Bicarbonate 10 mM, EDTA disodium salt 0.1 mM) and then washed several time with PBS. PBMCs samples were stored in liquid nitrogen until use. Blood was also collected at the same time points for sera recovery. Finally, animals from herd 1 and 2 were culled at 28 days post boost (dpb), wheras animals from herd 3 were culled at 14 dpb.

**Table 1:** Babraham's pigs immunization plan.

**Immunization**	**Animals**	**1st dose**		**2nd dose**	
		**Antigen**	**Route**	**Antigen**	**Route**
Strategy 1	Cohort 1				
	B557, B563, B568	SpH1N1	IM	SpH1N1	IM
	B558, B564, B570	Gripovac3	IM	Gripovac3	IM
Strategy 2	Cohort 2				
	1, 2	SpH1N1	IM	SpH1N1	IM
	CTRL	None	None	None	None
Strategy 3	Cohort 3				
	B625, B650	SpH1N1	IM	S-Flu	IN

*Two dose of the IAV were administered within a 28 days interval*.

### Culture Medium

Culture medium was RPMI-1640 medium with glutamax-I and 25 mM hepes (Life technologies) –emented with penicillin/streptomycin (Life technologies), and 10% heat-inactivated pig serum (Life technologies) or fetal calf serum (Gemini).

### Peptides

Pools 1–3 represented the entire NP (GenBank: AFG72805.1) and M1 (GenBank: AFG72802.1) protein sequences of human IAV strain A/Panama/2007/1999 (H3N2) fused with the amino acid sequence GGGPGGG. Pool MSP represented the Malaria Merozoite Surface Protein-1 was used as irrelevant peptide pool. Pools 1–3 and MSP were constituted by 80 peptides (numbered 1–80) with length between 17 and 20 amino acids and overlapping 10 amino acids. Pools A-D were pools of 6–8 peptides from pool 2. The amino acid identity of NP and M1 proteins between A/Panama/2007/1999 (H3N2) and SpH1N1 was 90 and 92% respectively; thus they are different enough to be considered heterologous (<97% sequence identity). Peptides were synthesized by the laboratory of proteomics & protein chemistry, Department of experimental & health sciences of the Pompeu Fabra University (Barcelona, Spain). All peptides were resuspended in DMSO and diluted in culture media to reach the desired concentration and stored at −80°C until use.

### Antigens Testing by *ex vivo* Tissues Stimulation

MDCK supernatants corresponded to the vehicle of SpH1N1 and it was used to determinate the background immune response produced as consequence of immunization with impure virus preparation. This antigen was obtained from mock infected MDCK cells by the same method used to propagate SpH1N1. Culture media was used to determinate background of the assays (Media). These and other antigens were diluted in culture media and when required DMSO. Where specified, DMSO was added to the stimulus diluted 500-fold to resemble the amount contained in peptide suspension. PBMCs were stimulated with the following amount of antigens per 5 × 10^5^ cells: 10^5^ TCID_50_ of SpH1N1, MDCK sup was diluted like virus, 2 × 10^5^ TCID_50_ of S-Flu, 1 μg of pools 1–3 and MSP and 0.2 μg of pools A-D or individual peptides. The restriction of the recalled immune responses was tested as previously described by (15). Anti SLA-I and SLA-II antibodies or isotype control antibodies were added to the *ex-vivo* recall stimulation of PBMCs as indicated in Table 3. The produced immune response was then quantified by IFNγ ELISPOT.

**Table 2:** Peptides representing the sequences of 43 and 44.

**Name**	**Sequence**	**Name**	**Sequence**
43a	NQQRASAGQISVQPTFSVQR	43m	ISVQPTFSVQRNL
43b	RASAGQISVQPTFSVQR	43n	QISVQPTFSVQRN
43c	AGQISVQPTFSVQR	43o	ISVQPTFSVQRN
43d	QISVQPTFSVQR	44a	SVQPTFSVQ
43e	SVQPTFSVQR	44b	SVQPTFSVQRNL
43f	VQPTFSVQR	44c	SVQPTFSVQRNLPF
43g	ISVQPTFSVQR	44d	SVQPTFSVQRNLPFEKS
43h	QISVQPTFSVQRNLPF	44e	SVQPTFSVQRNLPFEKSTVM
43i	ISVQPTFSVQRNLPF	44g	SVQPTFSVQRNLP
43j	QISVQPTFSVQRNLP	Sp44	SVQPTFSVQRNLPFERATIM
43k	ISVQPTFSVQRNLP	Irrelevant	NIKNESKYSNTFINNAYNMS
43l	QISVQPTFSVQRNL		

*The underline text represents the overlapping region of the 2 peptides*.

**Table 3:** Immunological reagents.

	**Clone**	**Subtype**	**Fluorophore**	**Brand**	**Amount**
**MHC BLOCKING**					5 × 10^5^ cells
**1°Antibodies**					
SLA-I	74-11-10	IgG2a	–	Kingfisher and in house	15 μg
SLA-II	MSA-3	IgG2b	–	Kingfisher and in house	15 μg
**Isotype controls**					
	W6/32	IgG2a	–	In house	NA

## IFNγ ELISPOT

Enzyme-linked immunosorbent spot (ELISPOT) plates were prepared with the capture antibody mouse anti-pig IFN-γ (Clone P2G10) (Becton Dickinson) as previously described (16). PBMCs were plated at 5 × 10^5^ cells per well and stimulated with antigens as indicated above. Pokeweed mitogen (PWM) 1 μg/ml or Concanavalin A (ConA) 10 μg/ml (Sigma) were used to stimulate 10^5^ PBMC as positive controls of the assay. Plates were cultured overnight at 37°C and 5% CO_2_. Spots were revealed by using a biotinylated mouse anti-pig IFNγ (Clone PTC11) (Becton Dickinson) antibody as previously described (16). Finally, dark blue-colored immunospots were counted using the AID ELISPOT reader (AID Autoimmun Diagnostika). Stimulation Index (SI) was calculated as ratio between ISC values of peptides and background (Media).

### Prediction of SLA-II Epitopes

The NetMHCpan 3.2 (17) *pan* algorithm was used to predict SLA-II epitopes of the NP of A/Panama/2007/1999 (GenBank: AFG72805.1). The Babraham's pigs SLA-II alleles were used as previously described: SLA-DRB1^*^05:01 (IPD-MHC: SLA06022), SLA-DQA^*^01:03 (IPD-MHC: SLA05927) and SLA-DQB1^*^08:01 (IPD-MHC: SLA05978) (18); their amino acid sequences were set up for a customized prediction.

### Conservation Analysis of Epitopes in IAV

Non identical and full length sequences of the NP protein of IAV isolated in swine (*n* = 2,721), human (*n* = 3,482) and avian (*n* = 5,640) were retrieved from the Influenza Research Database (19) (through the web site at http://www.fludb.org) (as of January 13th, 2020). Sequences were aligned with MAFFT (20) version 7 (https://mafft.cbrc.jp/alignment/server/large.html); the alignment was inspected and curated by using Jalview (21) version 2.11.0. The region spanning the identified epitope sequences were used to generate a logo by Weblogo3 (22) version 3.7.4 (http://weblogo.threeplusone.com/).

### Data Analysis

Data were transformed and graphed with Prism v5.01 (GraphPad). Descriptive statistics including distribution images of results and tables were performed with SAS system V.9.1.3 (SAS institute). Non-parametric statistics analysis was performed with Wilcoxon test implemented from SAS software. Individual pigs were used as experimental unit. The significance level (*p*) was set at <0.05 with tendencies reported when *p* < 0.1.

## Results

### *Ex-vivo* IFNγ Recall Responses in Babraham's Pigs

**Figure 1:**
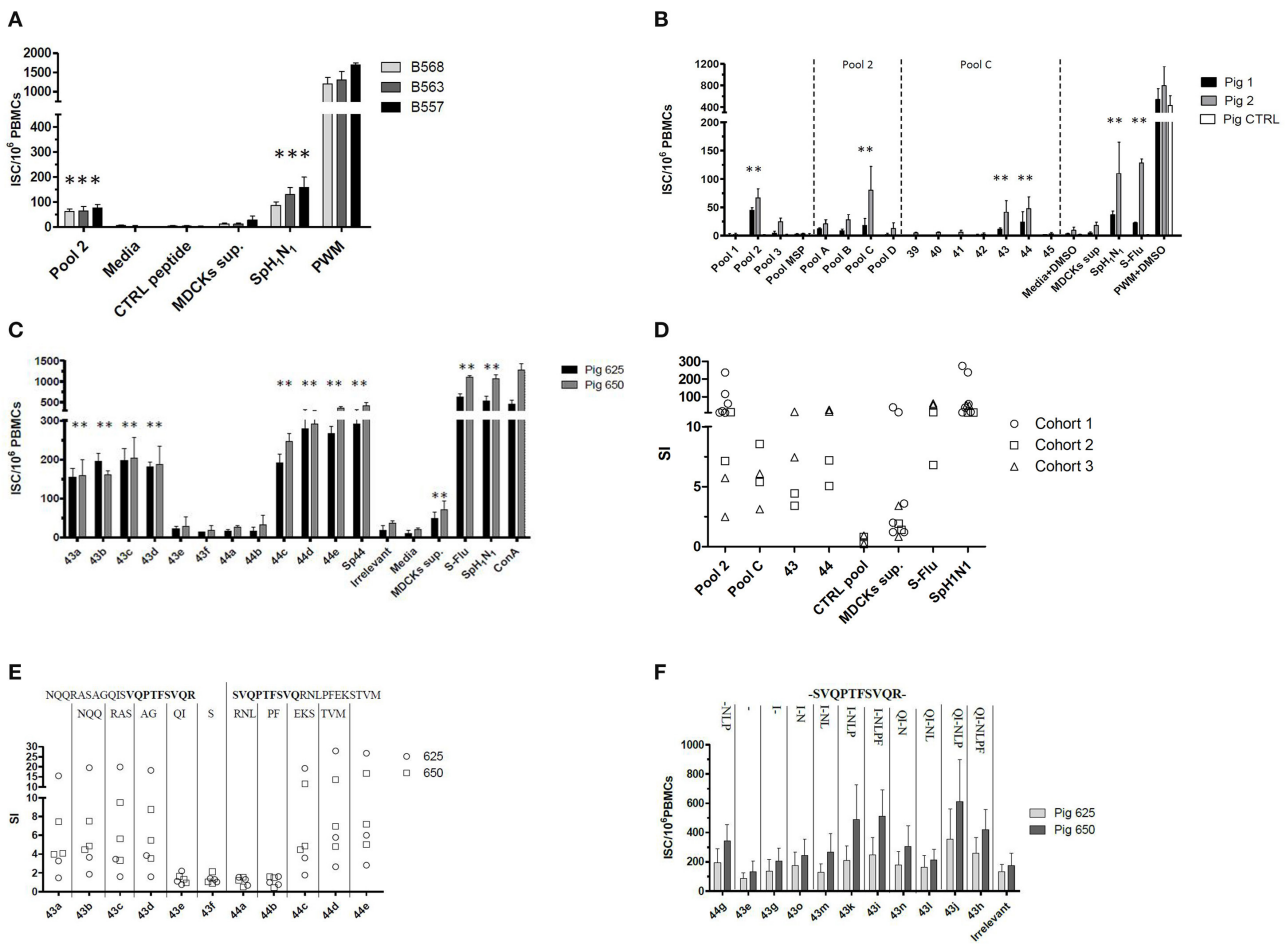
Empirical identification of T cells epitopes. A collection of peptides spanning the NP and M1 proteins of A/Panama/2007/1999 was used to stimulate *ex vivo* the PBMCs collected from Babraham's pigs. These were previously immunized using either **(A)** strategy 1 (i) SpH1N1 and (ii) Gripovac3 immunized pigs, **(B)** strategy 2 or **(C,E,F)** strategy 3. The recalled immune response was quantified by an IFNγ ELISPOT and calculated as number of interferon Gamma Secreting cells (ISC) per 10^6^ PBMCs **(A,B,F)**. The results obtained throughout the tests and cohorts were transformed as Stimulation Index (SI) and summarized in **(D,E)**; each dot represents the average value from a single assessment in a pig. The peptides were tested as pools (pools 1–3, pools **A–D**) and individually (39–45, 43a−44e, 44g−43h). The letters on the top of the graph in **(E)** represent the amino acids sequentially deleted from peptide 43a NQQRASAGQISVQPTFSVQR or 44e SVQPTFSVQRNLPFEKSTVM. In contrast the letter on the top of the graph in **(F)** represent the amino acid sequentially added from the peptide 43–44 overlapping region (in bold). The UV-inactived SpH1N1 and live S-Flu (H5) IAVs were used to recall a specific immune response in the immunized pigs. The MSP pool, the irrelevant peptide, the media either supplemented or not with DMSO were used to evaluate the non-specific response of the testing system. The MDCK sup was used to evaluate the non-specific immune response against the whole virus and in particular the derived from the replication system adopted. Finally, the PWM either supplemented or not with DMSO and the ConA were used to check the responsiveness of the PBMCs used in the assay. Statistically significant differences in **(A–C)** are indicated by asterisk (Wilcoxon signed-rank test; *p* < 0.05). Figures are representative of three independent experiments.

Babraham's pigs (Cohorts 1–3) were sequentially and differently administered with attenuated live and/or inactivated IAV antigens; the immunization strategy was set up and tested empirically with the aim of improving the *ex vivo* IFNγ secretion response against IAV. The tissues collected from these pigs were then tested *ex-vivo* to evaluate the immunization performances and also to identify and characterize T cell epitopes of IAV. The immunizations strategies primed an IFNγ secreting response against the live (S-Flu) or inactive (SpH1N1) whole virus when tested by ELISPOT (Figures 1A–D). Viruses used to immunize the pigs were genetically divergent (13) but despite this, they were able to produce a cross-reacting IFNγ response. In particular, S-Flu belonged to a completely different lineage of IAV compared to SpH1N1 or Gripovac3 antigens; however, it was able to recall *ex-vivo* an IFNγ response on PBMC collected from pigs immunized with SpH1N1 (Cohort 2) (Figure 1B).

### Identification of an Antigenic Regions in the M1 and NP Proteins of IAV

The strain A/Panama/2007/1999 (H3N2) is an IAV belonging to a different subtype and lineage compared to the antigens used for the immunization of pigs. In particular, its M1 and NP proteins shared between 92.06–95.63% and 89.76–91.97% of the amino acid sequences, respectively, with them. To identify the antigenic regions able to produce a cross reacting IFNγ response, a collection of overlapping peptides representing the two proteins of A/Panama/2007/1999 were tested *ex vivo* by ELISPOT. All peptides were initially tested in pools (pools 1–3) and subsequently dissected into smaller subsets up to identify those individual peptides inducing a statistically significant IFNγ secretion response. Pool 1 and 2 represented the NP protein whereas pool 3 represented the M protein of IAV. The assay was initially performed by using the PBMCs purified from Babraham's pigs immunized with the heterologous antigens SpH1N1 and Gripovac3 (Cohort 1 and Cohort 2). The immunodominant response was obtained by pool 2 being the other pools not responding (Figures 1A,B,D). Further analysis of peptides included in pool 2 identified peptide 43 and 44 as immunodominant (Figures 1B,D).

To identify the shortest amino acid sequence inducing an IFNγ secretion response, a new set of peptides with sequential deletion of the end terminals regions was designed from the peptides 43 and 44 sequences (Table 2). This set of peptides was then tested by ELISPOT using PBMCs from Babraham's pigs immunized with the heterologous antigens SpH1N1 and S-Flu (Cohort 3). The shortest identified immunogenic regions (Figures 1C,E) were represented by peptides 43d and 44c which were then further dissected with a new set of peptides designed with the same strategy adopted before (Table 2). Moreover, IFNγ secretion responses induced by the region spanning the entire sequence of peptide 43d and 44c was also tested (Figure 1F). Unfortunately, the potency to carry out a statistical analysis was extremely low. Thus, it was almost impossible to find significant differences among peptides. Peptide 43g and 44g were the shortest immunogenic regions identified in this study. Notably, the removal of I at the left terminal of peptide 43g and NLP in 44g was fundament to maintain the immunogenic properties of each peptide. The effects of the right and left terminal end amino acids of peptides 43g and 44g were not further characterized. Moreover, the following effects have been observed: Peptide 43k and 43i fulfilled the hypothesis that the sum of 2 epitopes (44g and 43g) increased responses compared to those observed by stimulation with each one alone. Peptide 43j showed that simultaneous addition of Q and P amino acids enhance IFNγ response of peptide 43k. Finally, peptide 43h suggested that F partially suppress responses of 43J.

Peptide 43g and 44g corresponded, respectively, to regions NP_406−416_ (ISVQPTFSVQR) and NP_407−419_ (SVQPTFSVQRNLP) of A/Panama/2007/1999. Conservation of these regions in the IAV used as immunization antigens in this study was inspected. Region NP_406−419_ was mutated in H3N2 virus contained in Gripovac3 (I406V) and in the S-Flu (V408I) whereas it was completely conserved in the H1N1 and H1N2 strains of Gripovac3 and also in SpH1N1. Therefore, the Babraham's pigs were immunized at least once with an antigen containing the same sequence of the IAV epitopes identified in this study.

### Identification of the SLA Class Restriction

**Figure 2:**
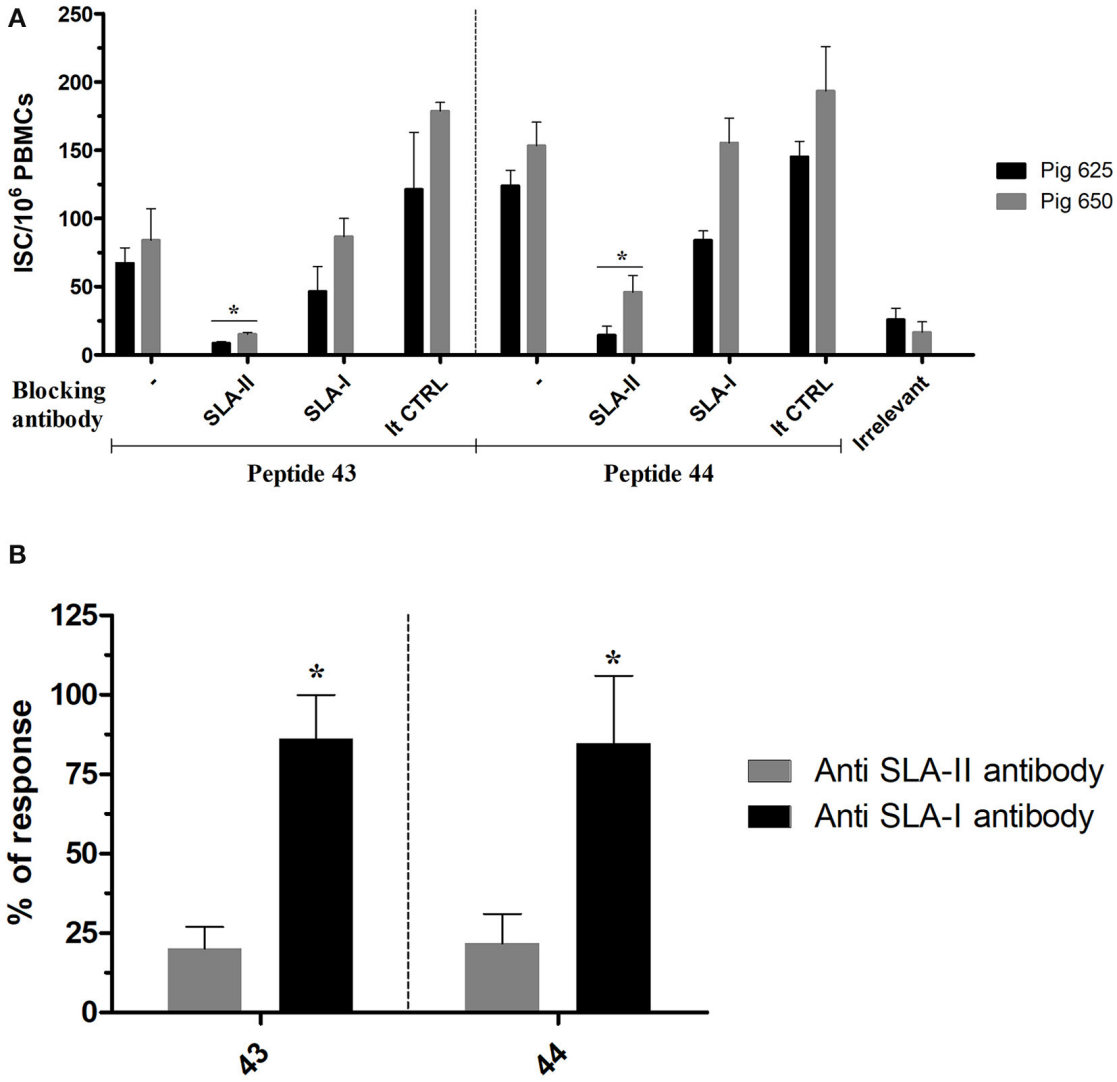
SLA class restriction of the identified immunogenic regions. The peptides 43 and 44 were used to recall *ex-vivo* an immune response in PBMC collected from pigs immunized with IAV (cohort 3) in presence of SLA-II and SLA-I specific antibodies. The response was then quantified by IFNγ ELISPOT and calculated as ISC/10^6^ PBMCs. The irrelevant peptide was used to evaluate the specificity of the peptide induced response. The isotype control antibodies were used to check the specificity of the restriction of the anti SLA antibodies. **(A)** The response of each tested pigs was represented as mean with sd; the asterisks indicate a statistically significant difference (Wilcoxon signed-rank test; *n* = 3; *p* < 0.05). **(B)** Percentage of the ISC/10^6^ PBMCs recalled in presence of anti SLA-antibodies compared to their absence. The asterisk indicates a statistically significant difference (Wilcoxon signed-rank test; inter assay *n* = 4; *p* < 0.05).

The SLA class restriction of NP_406−416_ and NP_407−419_ (contained, respectively, in peptides 43 and 44) was inferred by testing their immunogenicity in presence of either an anti SLA-I or SLA-II antibody. PBMCs from Babraham's pigs immunized with IAV were stimulated *ex-vivo* with the peptides in presence of either of the anti SLA antibodies which blocked the responses mediated by those receptors; the produced immune response was then quantified by an IFNγ ELISPOT. Responses induced by the tested antigens was inhibited by the anti SLA-II antibody with much greater magnitude compared to anti SLA-I antibody (Figure 2). In particular, the first inhibited 84 and 86% of peptides 43 and 44 recall responses, respectively; in contrast, <25% of the responses were blocked by the anti SLA-I antibodies.

**Figure 3:**
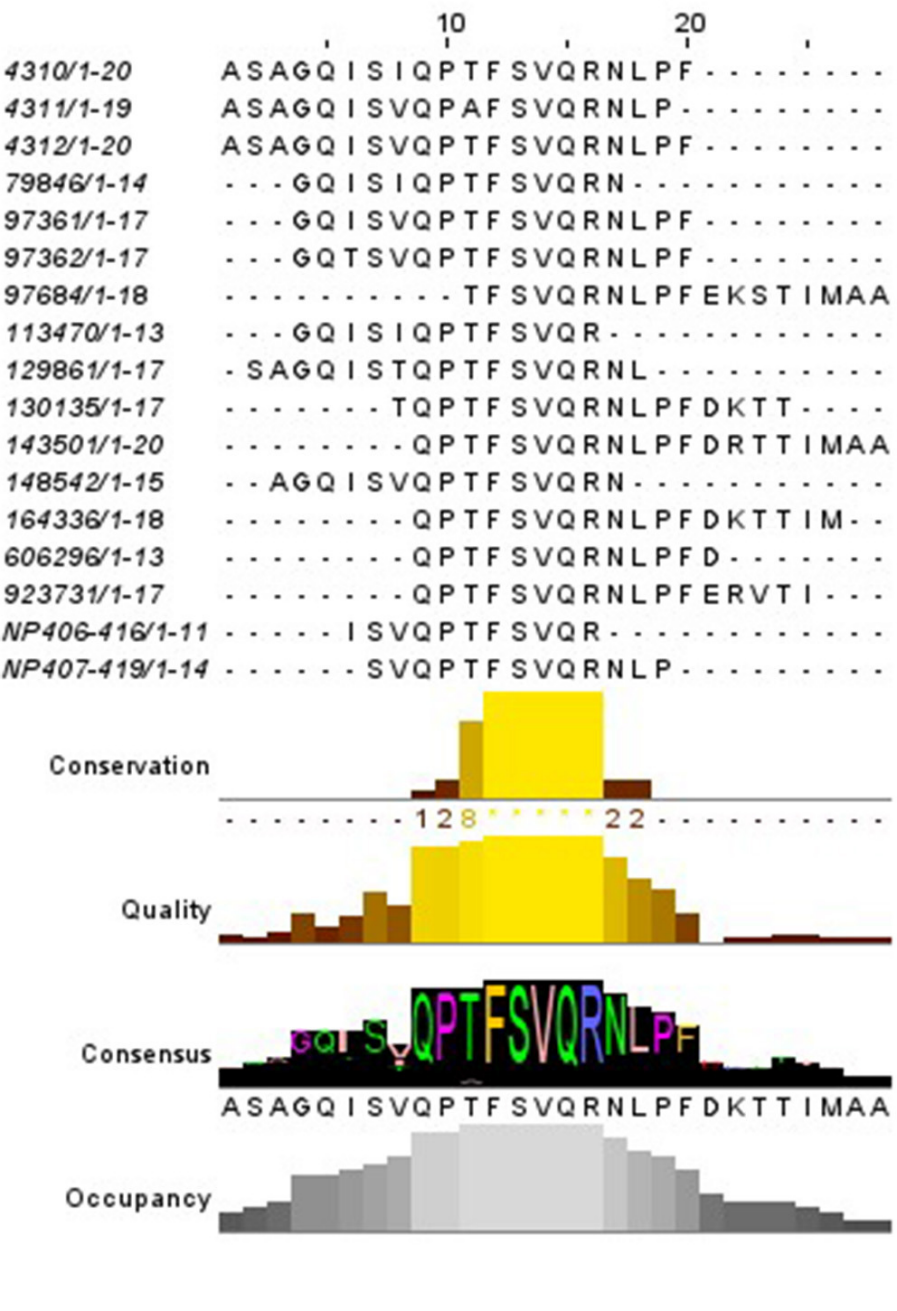
Alignment of the MHC-II epitopes similar to NP_406−419_ described. The red square indicates the region shared by most of the epitopes. The code on the left represent the IEDB reference.

The IEDB database was then inquired to investigate whether other MHC-II epitopes were described falling in the region NP_406−419_, regardless the species associated. For this purpose, a search for similar linear MHC-II restricted (blast 70%) epitopes was set up. The database contained 17 sequences of MHC-II restricted epitopes related to the NP_407−419_ of IAV deposited up to 2019 (Figure 3); notably, the human MHC-II HLA-DRB were the most related alleles, when this specific information was available.

The relationship of NP_406−416_ and NP_407−419_ with the Babraham's pigs SLA-II alleles was tentatively inferred by *in-silico* prediction. As there are no open available algorithm of prediction designed specifically for pigs a *pan* algorithm was used for such purpose The Babraham's pigs SLA-II alleles (DRB1 and DQ) and the NP (A/Panama/2007/1999) sequences were set up in NetMHCpan 3.2. The resulting peptide sequences were ranked depending on their predicted affinities and then those sharing part of their sequence with NP_406−416_ and NP_407−419_ were retrieved. Notably, NP_412−420_ FSVQRNLPF and NP_406−414_ ISVQPTFSV were ranking, respectively, among the top 2% (strong binders) and 10% (weak binders) of the 9mer predicted peptides for the SLA-DRB1. In contrast, no similar epitope was found for the SLA-DQ.

The SLA-II allele restriction of the region NP_406−419_ was tentatively tested as described above but in presence of antibodies specific for the alleles DR and DQ to give support to the above predictions. The antibody specific for the SLA-II DR allele decreased up to a maximum of 80% the responses of peptides 43i and 43j (Figure 1 SI). The assay had low power as it was performed just two times in a single pig and thus it deserved further studies.

### Conservation of NP_406−416_ and NP_407−419_ Among IAV

**Figure 4:**
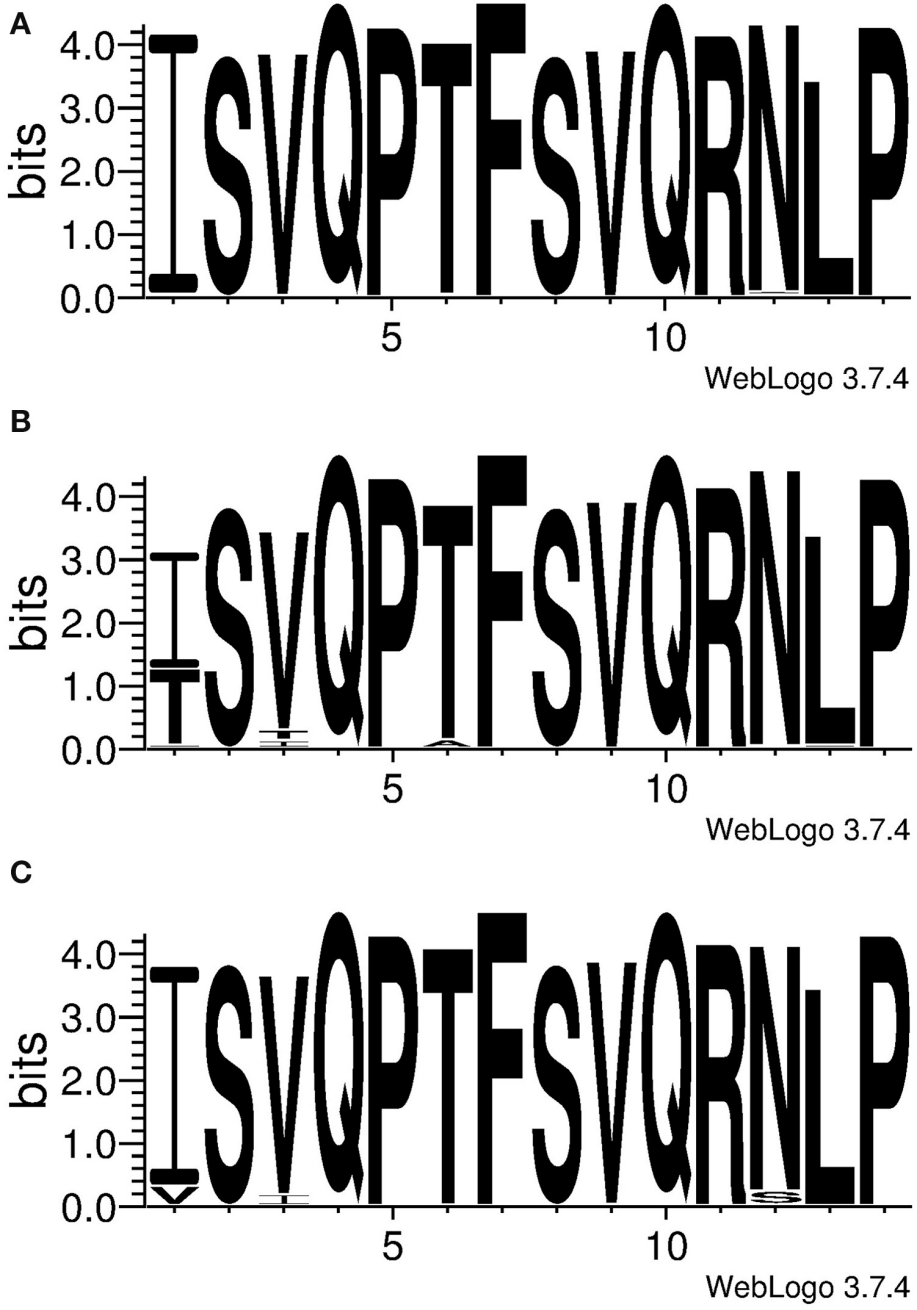
Conservation of NP_406−419_, ISVQPTFSVQRNLP. The logo was generated using unique NP sequences of IAV isolated from **(A)** swine **(B)** human or **(C)** avian species. Letters represent the amino acid code and their height is proportional with the frequency.

Conservation of the identified epitopes was inspected in the IAV isolated from the three major host species (swine, human and avian specie). For this purpose, a collection of non-redundant full NP sequences of IAV was retrieved from a public domain database and aligned; the generated database was therefore meant to contain unique variants of IAV. Then the variability at the region NP_407−419_ were presented as logo in Figure 4. SwIV showed a high conservation of the entire sequence; in particular, NP_417_ (98.09%) and NP_406_ (98.97%) were the least conserved positions, whereas the rest of the sequence was conserved in more than 99% of SwIV strains (Table 1 SI).

**Figure 5:**
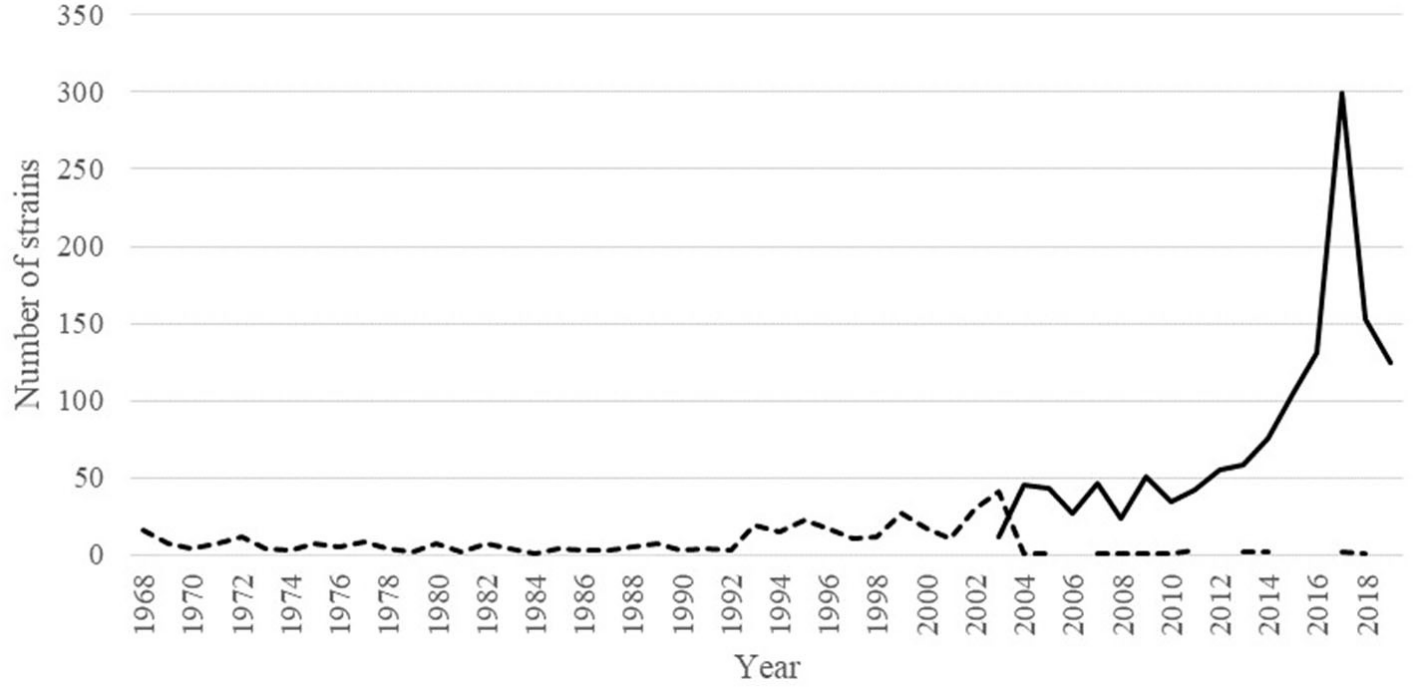
Dynamic of the I406T amino acid mutation in sequenced H3N2 IAV variants since their introduction in humans. Dotted line represents the number of strains having I406 whereas the continuous line represents the number of strains having T406 in their NP protein.

Human IAV showed an inconsistent conservation throughout the NP_407−419_ sequence. Position NP_406_ was conserved in just 59.82% of the inspected strains whereas 38.71% of the rest of them showed the mutation I406T (Table 1 SI). Notably, 98.04% (*n* = 1,327) of the strains bearing the mutation I406T were belonging to the H3N2 subtype (Table 2 SI); this was not a bias due to an over representation of the H3N2 subtype in the database as they constituted the 48.7% of the strains, whereas the H1N1 constituted the 43.5%. The number of strains containing Isoleucine or Threonine amino acids at position NP_406_ were distributed throughout the last 51 years (since the introduction of H3N2 IAV in humans) depending on their isolation time. Figure 5 showed that I406T mutation started to be detected since the 2000 and its presence increased dramatically up to 2017. Other least conserved positions were, respectively, NP_408_ (91.67%), NP_411_ (96.41%) and NP_418_ (98.48%) whereas the rest were conserved in more than 99% of the inspected human IAV strains (Table 1 SI).

The avian IAV also showed the lowest conserved position at NP_406_, however, unlike human IAV, Isoleucine was found in a higher proportion of strains (91.79%). This position showed a high preference for the mutation I406V (8.10%). Positions NP_417_ and NP_408_ were conserved, respectively, in 94.38 and 95.20%, whereas, the rest of position was conserved in more than 99% of avian IAV (Table 1 SI).

Based on the conservation findings, the cross-reactive propriety of the identified region NP_406−419_ was tentatively tested in a completely different experiment. PBMCs samples were retrieved from a previous study conducted in Babraham's pigs in which they were immunized with a non-adjuvated whole A(H1N1)pdm/09 influenza vaccines licensed for humans (16). This NP segment of this virus derived from the “classical” SwIV which is a different lineage compared to the immunizing antigens used above. Results showed that peptides 43i and 43j were able to recall an IFNγ secretion responses in one of the immunized pigs whereas the rest and the mock-vaccinated pigs were not responding (Figure 2 SI). However, the assay had low power as it was repeated two times and thus it deserved further studies.

## Discussion

The CD4^+^ T cell response is considered of great importance in immune response against IAV; it provides help to CD8^+^ T cell and B cell responses but it also directly controls virus spread by means of cytotoxic effects on infected cells [Reviewed in (23)]. Novel strategies of rational design of IAV vaccines are therefore considering to modulate T cell responses including not only CD8 but also the CD4 subsets (4).

In swine, few SLA-II epitopes of IAV have been identified, as compared to HLA epitopes. In the present study, SLA-II epitopes of IAV were identified in pigs by using an empirical approach. Babraham's pigs were used as model to identify T cells epitopes because their syngeneic MHC loci which aids identification of SLA restriction of the identified epitopes. Previous studies demonstrated that immunization strategies based on the administration of whole inactive virus (WIV) formulated with adjuvant in pigs were able to produce an IFNγ or proliferative cell mediated response in peripheral blood involving T helper subsets (8, 16, 24). Moreover, the generated immunity was also able to respond to heterologous IAV strains (24).

Similar results were obtained in this study by boost immunization of Babraham's pigs with live and/or inactivated whole IAV antigens; in particular, the latter was constituted by an *in-house* UV-inactivated IAV formulated with adjuvant or a commercially available vaccine which was licensed by that time in pigs. The adopted immunization strategies produced a specific IFNγ immune response in the Babraham's pigs; this was recalled *ex vivo* by stimulating PBMC with homologous and even heterologous IAV antigens and it was detected by means of an IFNγ ELISPOT assay. This study is therefore supporting previous finding that influenza vaccines for pigs based on WIV and formulated with adjuvant are able to generate T cell responses with IFNγ secreting effector functions; moreover, it supports the generation of T cell responses with cross reactivity against viruses of different linages For this purpose, a collection of peptides spanning the entire length of the NP and M1 proteins was tested *ex-vivo* by using an IFNγ ELISPOT assay on PBMC harvested from Babraham's pigs previously immunized with IAV. The adopted immunization and screening strategies permitted to successfully identify two overlapping immunogenic regions: NP_406−416_ (ISVQPTFSVQR) and NP_407−419_ (SVQPTFSVQRNLP). Despite sharing most of their sequences, they are considered different epitopes because the non-overlapping positions (NP_406_ and NP_417−419_) were critical to maintain their respective immunogenic proprieties. NP_406−416_ (ISVQPTFSVQR) and NP_407−419_ (SVQPTFSVQRNLP are the shortest epitopes tested in this study, the effect of the removal of the amino acid at the right terminal of NP_406−416_ or both end terminals of NP_407−419_ were not tested. The MHC restriction of the identified regions was inferred by blocking IFNγ recall responses with class specific antibodies, a strategy previously adopted in pigs by other authors (15). Peptides containing the identified regions showed specificity for the SLA-II; therefore, no SLA-I epitope was identified in this study.

A previous study demonstrated that immunization with inactive IAV antigens is able to produce a Cytotoxic T lymphocytes response against the whole virus in Babraham's pigs, although this was detected as proliferative response (16); moreover, SLA-1 of the same pig lineage were identified in the NP protein of IAV by means of a strategy involving long-term *in vitro* pig T-cell culture and cloning (25). Therefore, the missed identification of SLA-I epitopes might have been due to differences in the immunization and/or screening strategy.

The IDEB database contained just three M1 and one NP related epitopes restricted to the SLA-II. These were discovered by previous authors using *in silico* prediction followed by *ex-vivo* IFNγ ELISPOT testing (8). In the present study, no SLA-II epitope producing an IFNγ recall response was identified in the M1 protein whereas the epitopes found in the NP were located in a different region compared to the one previously published (NP_253−277_). These differences might have been due to the allele diversity between the cohorts of pigs of the two studies, the SLA low resolution alleles found by the authors were different compared to Babraham's pigs.

Despite the absence of similar epitopes in pigs, several human MHC-II epitopes were previously identified by other authors. Notably, most of those were restricted to HLA-DRB1 alleles and raised the question whether this affinity might have been conserved in pigs as well. The pan algorithm NetMHC II 3.2 was used to predict epitopes of the NP of A/Panama/2007/1999 associated to the Babraham's pig SLA-II allele. Notably, two 9mer epitopes were predicted for the SLA-DRB1, NP_412−420_ FSVQRNLPF and NP_406−414_ ISVQPTFSV; these epitopes were partially contained in the sequences of epitopes NP_406−416_ and NP_407−419_ identified in this study. A proof of concept test conducted with low power showed that the immunogenic response produced by peptides containing the region NP_406−416_ was not restricted to the DQ allele but instead by the DRB allele. Therefore, these results support the idea that those epitopes might be associated to the SLA-DRB1 allele in Babraham's pigs, however, further study are required to validate the *in silico* prediction.

Different functionally polarized subpopulations have been established in studies with murine and human CD4^+^ T cells and just a part of these were elucidated in pigs (26). This is a mayor limitation in the SLA-II epitope discovery strategies in pigs whereby cytokines are biomarker of cells responsiveness. In the present study, IFNγ was used as functional marker to quantify the recall response to the overlapping IAV peptide collections in peripheral blood. This cytokine is generally associated in pigs to the Th1 subset of the CD4^+^ T cells (26). Single and double producer of IFNγ and TNFα dominated the response of CD4^+^ T cells in blood and other tissues during an IAV infection in pigs and thus they were suggested to be important in its control (6). Therefore, the immunogenicity of NP_406−416_ and NP_407−419_ during infection and their involvement in protection deserve further studies. The encounter SLA-II epitopes might be also helpful under other effector functions. The phenotype of CD4^+^ T cells depends on the “cytokine milieu” (27) and thus this might be rationally modulated to a specific phenotype involved in other T helper functions.

Sequence conservation of epitopes is an important aspect to take into account during the rational design of an IAV vaccine. The use of conserved epitopes could contribute to the breadth of protection conferred by the vaccine. The SLA-II T cell epitopes identified in this study were hailing from the human IAV strain A/Panama/2007/1999 (H3N2) and despite this, they showed to be able to induce a cross reacting response in pigs immunized with IAV of completely different lineages. This fact was due to the complete conservation of their amino acid sequences among the antigens used in this study. NP and M1 proteins are codified by the internal cassette of genes of IAV which is by far more conserved compared to the HA and NA coding genes. Notably, the internal cassette of the European swine lineages of the H3N2 and H1N2 subtypes is even more conserved because it was shared by the H1N1 avian like lineage through reassortment events. The analysis of swine IAV showed an impressive conservation of the NP_406−419_ sequence which suggests this region might be an optimal candidate for development of a broad reacting vaccine for pigs. Nevertheless, conservation of the sequence in other mayor species is also an important factor to take into account for the design of a broad protecting vaccine. IAV has the ability to cross the specie barrier whereas pigs have the ability to act as a “mixing vessel” and also to perpetrate other species lineages. Human and avian species are the other two major known host of IAV; these might act as reservoir of novel variants that might escape from the pig immune system recognition of NP_406−419_. Conservation analysis showed that this sequence was less conserved in human and avian IAV; in particular, the site NP_406_ showed the lowest conservation in both lineages, especially in the human. Notably, results showed that the mutation I406T is highly frequent in human IAV lineage and it was particularly associated with H3N2 strains circulating since the ‘00s. During the same period a transition to a novel antigenic cluster [A/Fujian/411/2002 (FU02)] linked to a persistent reassortment event was observed (28). Considering the common ancestor between the avian IAV and other species lineages it was not surprising to find the conserved amino acid Isoleucine at the position NP_406_; however, results showed that the divergent evolution of IAV in human hosts is apparently changing permanently its composition. NP_406_ was a critical position to maintain the immunogenic properties of the epitope NP_406−416_ and actually its removal disrupted the capacity of the T cell epitope to recall an IFNγ response in the immunized pigs. The effects of mutations at NP_406_ on the immune proprieties of the T cell epitope are unknown; because of the variability and evolution of the IAV, this position deserve further studies to evaluate its antigenic tolerance.

The overall high conservation of NP_406−419_ is probably due to its functional importance in the NP protein. The region is localized into a flexible tail-loop (residues 402–428) which mediate the NP-NP oligomerization (29). In this region, positions like the R416 can be found which is critical for the functionality of the protein (29); therefore, this region is expected to be less permissive to mutations. Thus, region NP_406−419_ can be considered an optimal candidate for vaccines research due to the presence of T cells epitopes and its high conservation.

Considering the high conservation in the epitope, a proof of concept test was conducted with low power to evaluate the cross reactivity of the identified immunogenic region NP_406−416_. Results showed that this region showed to be immunogenic in pigs even in a different immunity scenario involving the A(H1N1) pdm/09 influenza vaccines and thus support its validity as candidate for further studies.

## Data Availability Statement

The raw data supporting the conclusions of this article will be made available by the authors, without undue reservation.

## Ethics Statement

The animal study was reviewed and approved by The Pirbright Institute ethics committee.

## Author Contributions

MM and BC: funding acquisition. MM: supervision and conceptualization. MB, SM, JH, ER, BVC, EL, SM-T, LF, and ET: investigation and methodology. MB and MM: writing-original draft. All authors contributed to the article and approved the submitted version.

## Conflict of Interest

SM-T was employee of Innovex Therapeutics S.L. The remaining authors declare that the research was conducted in the absence of any commercial or financial relationships that could be construed as a potential conflict of interest.
