# Supplementary material for: Identification of a Newly Conserved SLA-II Epitope in a Structural Protein of Swine Influenza Virus
Source: Front Immunol. 2020 Sep 16;11:2083. doi: 10.3389/fimmu.2020.02083 (PMC7524874; doi:10.3389/fimmu.2020.02083)
Supplement: Supplementary file 1 [file Data_Sheet_1.PDF]

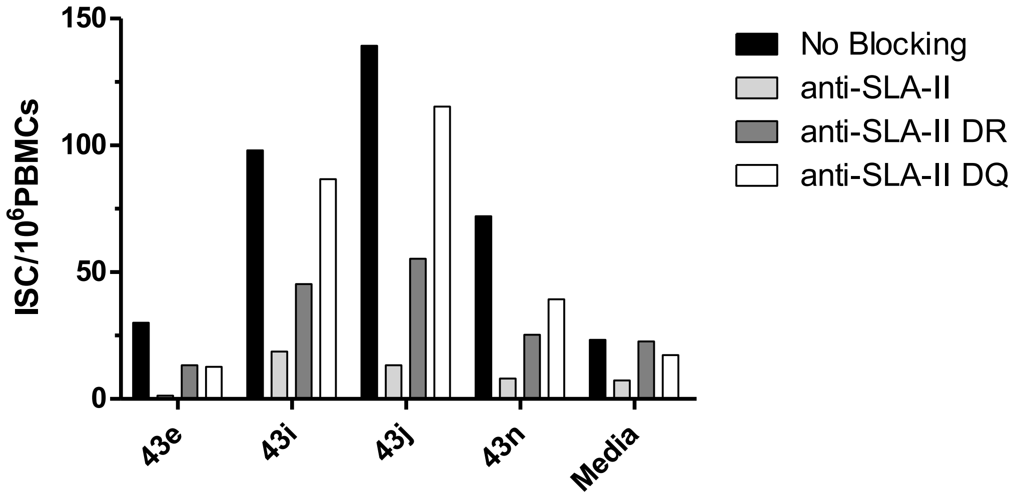

Figure 1 SI. SLA-II allele restriction of the identified immunogenic regions. Peptides were used to recall *ex-vivo* an immune response in PBMCs collected from pig 650 immunised with IAV (cohort 3) in presence of an SLA-II class specific and SLA-II DR (Clone 1D2) DQ (Clone 2E12) allele specific antibodies. The response was then quantified by IFNγ ELISPOT and calculated as ISC/10^6 PBMCs. The graph represent the results from a single experiment and thus lack of enough statistical power.

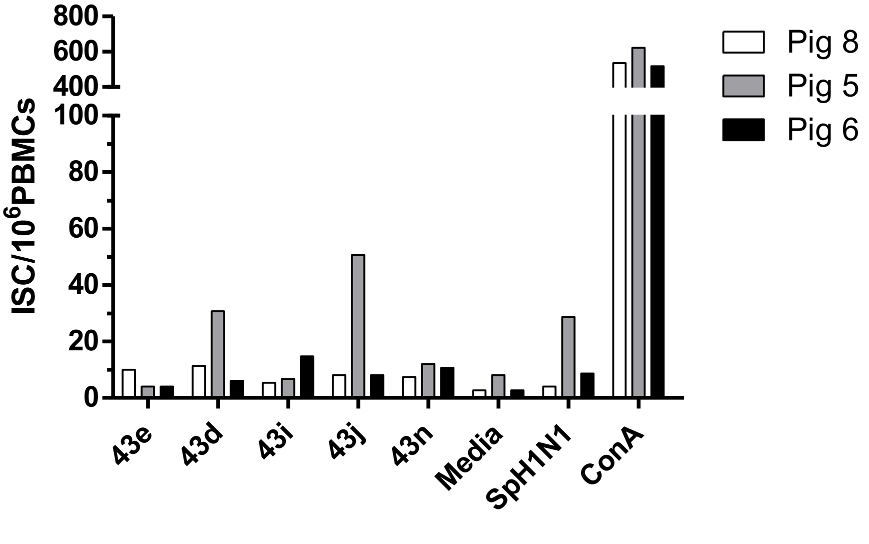

Figure 2 SI. Cross reactivity of the identified immunogenic regions. The peptides identified in this study were used to recall *ex-vivo* an immune response in PBMCs. These were collected during a previous study from pig immunised either with a non-adjuvated whole A(H1N1)pdm/09 influenza vaccines licensed for humans (pigs 5 and 6) or mock-vaccinated (15). The response was then quantified by IFNγ ELISPOT and calculated as ISC/10^6 PBMCs. The graph represent the results from a single experiment and thus lack of enough statistical power.

TABLE SI1 Conservation of $NP_{406\text{-}419}$ in swine, human and avian IAV strains.

Position	406	407	408	409	410	411	412	413	414	415	416	417	418	419
	I	S	V	Q	P	T	F	S	V	Q	R	N	L	P
<u>Swine</u>														
Percentage	98.97	100.00	99.38	99.96	99.93	99.12	100.00	99.96	99.89	100.00	100.00	98.09	99.67	100.00
Variants	V 1.03%		I 0.51%<br>M 0.07%<br>T 0.04%	K 0.04%	A 0.07%	A 0.84%<br>S 0.04%		F 0.04%	I 0.11%			S 1.28%<br>T 0.29%<br>D 0.22%<br>H 0.04%<br>K 0.04%<br>G 0.04%	I 0.29%<br>F 0.04%	
<u>Human</u>														
Percentage	59.82	99.97	91.67	99.97	100.00	96.41	99.94	99.94	99.94	99.97	99.94	99.14	98.48	99.94
Variants	T 38.71%<br>V 1.46%	N 0.03%	T 4.77%<br>I 3.42%<br>M 0.09%<br>S 0.06%	E 0.03%		A 3.56%<br>N 0.03%	I 0.06%	L 0.06%	I 0.03%<br>M 0.03%	R 0.03%	K 0.03%<br>P 0.03%	S 0.49%<br>T 0.26%<br>D 0.09%<br>B 0.03%	I 1.41%<br>H 0.06%<br>F 0.03%<br>J 0.03%	S 0.06%
<u>Avian</u>														
Percentage	91.79	99.98	95.20	100.00	99.98	99.72	99.95	99.95	99.43	99.95	99.96	94.38	99.86	99.98
Variants	V 8.10%<br>T 0.09%<br>L 0.02%	R 0.02%	I 4.73%<br>L 0.04%<br>M 0.02%<br>T 0.02%		R 0.02%	A 0.23%<br>S 0.04%<br>P 0.02%	L 0.05%	T 0.04%<br>P 0.02%	I 0.57%	H 0.05%	G 0.04%	S 5.14%<br>T 0.27%<br>D 0.16%<br>G 0.04%<br>K 0.02%	I 0.07%<br>F 0.04%<br>H 0.02%<br>P 0.02%	S 0.02%

TABLE SI2 Distribution of the amino acid variants at position NP_{406} among the main subtype of the human IAV database studied.

Amino acid	H1N1		H3N2		Others		Total
	n	%	n	%	n	%	n
I	1502	111	368	27,3	213	15,8	2083
T	4	0,3	1327	98,4	17	1,26	1348
V	7	0,52	0	0	44	3,26	51
Total	1513	43,5	1695	48,7	274	7,87	3482
